# An auxin responsive *CLE* gene regulates shoot apical meristem development in *Arabidopsis*

**DOI:** 10.3389/fpls.2015.00295

**Published:** 2015-05-01

**Authors:** Hongyan Guo, Wei Zhang, Hainan Tian, Kaijie Zheng, Xuemei Dai, Shanda Liu, Qingnan Hu, Xianling Wang, Bao Liu, Shucai Wang

**Affiliations:** Key Laboratory of Molecular Epigenetics of MOE and Institute of Genetics and Cytology, Northeast Normal UniversityChangchun, China

**Keywords:** auxin, peptide hormone, *OsCLE48*, *CLV3*, apical meristem, *Arabidopsis*, *Oryza sativa*

## Abstract

Plant hormone auxin regulates most, if not all aspects of plant growth and development, including lateral root formation, organ pattering, apical dominance, and tropisms. Peptide hormones are peptides with hormone activities. Some of the functions of peptide hormones in regulating plant growth and development are similar to that of auxin, however, the relationship between auxin and peptide hormones remains largely unknown. Here we report the identification of *OsCLE48*, a rice (*Oryza sativa*) *CLE* (*CLAVATA3/ENDOSPERM SURROUNDING REGION*) gene, as an auxin response gene, and the functional characterization of *OsCLE48* in *Arabidopsis* and rice. *OsCLE48* encodes a CLE peptide hormone that is similar to *Arabidopsis* CLEs. RT-PCR analysis showed that *OsCLE48* was induced by exogenously application of IAA (indole-3-acetic acid), a naturally occurred auxin. Expression of integrated *OsCLE48p:GUS* reporter gene in transgenic *Arabidopsis* plants was also induced by exogenously IAA treatment. These results indicate that *OsCLE48* is an auxin responsive gene. Histochemical staining showed that *GUS* activity was detected in all the tissue and organs of the *OsCLE48p:GUS* transgenic *Arabidopsis* plants. Expression of *OsCLE48* under the control of the *35S* promoter in *Arabidopsis* inhibited shoot apical meristem development. Expression of *OsCLE48* under the control of the *CLV3* native regulatory elements almost completely complemented *clv3-2* mutant phenotypes, suggesting that OsCLE48 is functionally similar to CLV3. On the other hand, expression of *OsCLE48* under the control of the *35S* promoter in *Arabidopsis* has little, if any effects on root apical meristem development, and transgenic rice plants overexpressing *OsCLE48* are morphologically indistinguishable from wild type plants, suggesting that the functions of some CLE peptides may not be fully conserved in *Arabidopsis* and rice. Taken together, our results showed that *OsCLE48* is an auxin responsive peptide hormone gene, and it regulates shoot apical meristem development when expressed in *Arabidopsis*.

## Introduction

Auxin regulates many aspects of plant growth and development, including apical dominance, organ formation, lateral root formation, root and stem elongation, and vascular development (Davies, 1995). It is very likely that auxin regulated plant growth and development is initiated by the activation of auxin response genes by locally increased auxin concentration (Chapman and Estelle, 2009).

Activation of auxin response genes is controlled by two different groups of transcription factors, auxin response factors (ARFs) and Aux/IAA proteins. When the cellular auxin level is low, Aux/IAA proteins are dimmerized with ARFs, thus inhibit the expression of auxin response genes. When the auxin level is elevated, auxin binds to TIR1 auxin receptor, leading to the activation of TIR1, and eventually the degradation of Aux/IAA proteins by 26S proteasome, result in the activation of auxin response genes by ARFs (Dharmasiri et al., 2005; Kepinski and Leyser, 2005; Guilfoyle and Hagen, 2007; Tan et al., 2007; Hayashi, 2012).

Several different gene families including *Aux/IAAs*, *GH3s*, and *SAURs* have been identified as auxin response genes (Hagen and Guilfoyle, 2002). Expression of some other genes such as *ASL/LBD* (*LATERAL ORGAN BOUNDARIES DOMAIN/ASYMMETRIC LEAVES2-LIKE*) is also induced by auxin (Lee et al., 2009; Coudert et al., 2013). However, considering that auxin regulates almost every aspect of plant growth and development, it is likely that large numbers of auxin response genes remain unidentified (Kieffer et al., 2010).

Peptide hormones are peptides with hormone activities. Peptide hormones regulate many cellular processes in animals, bacteria and yeast (Edlund and Jessell, 1999). It has been believed that plants do not produce peptide hormones, until the identification of Systemin, a 18 amino acids peptide, as a signal molecule in wounding response in tomato (Pearce et al., 1991). So far nearly 20 plant peptide hormones have been identified (Germain et al., 2006; Hashimoto et al., 2008; Katsir et al., 2011; Leasure and He, 2012).

Plant peptide hormones are encoded by genes with small open reading frames (ORFs), and can be classified into two different groups: secreted and non-secreted peptide hormones (Hashimoto et al., 2008; Katsir et al., 2011; Matsubayashi, 2011). Precursors of secreted peptide hormones contain an N-terminal signal peptide, which facilities protein transport and will be removed during post-transcriptional modifications. On the other hand, non-secreted peptide hormones do not contain signal peptides (Hashimoto et al., 2008; Katsir et al., 2011; Matsubayashi, 2011).

Non-secreted plant peptide hormones include Systemin, Enod40 (Early Nodulin 40), POLARIS (PLS), ROT FOUR LIKE (RTFL), amd BRICK1 (BRK1; Charon et al., 1997; Germain et al., 2006; Hashimoto et al., 2008). Non-secreted peptide hormones are involved in the regulation of plant growth and development, and plant response to enviromental stresses. For example, PLS regulates root growth and vascular development (Casson et al., 2002), RTFL peptides DEVIL (DVL1) and ROTUNDIFOLIA4 (ROT4) regulate leaf and fruit development (Narita et al., 2004; Wen et al., 2004), IDL peptides regulate floral organ abscission (Butenko et al., 2003; Cho et al., 2008; Stenvik et al., 2008), and Systemin invloves in the regulation of plant stress response (Pearce et al., 1991, 2001; Constabel et al., 1998).

More than 10 different secreted peptide hormones have been identified in plants, including CLAVATA3/ENDOSPERM SURROUNDING REGION (CLE), EPIDERMAL PATTERNING FACTOR (EPF), ROOT MERISTEM GROWTH FACTOR (RGF)/CLE LIKE (CLEL)/GOLVEN (GLV), INFLORESCENCE DEFICIENT IN ABSCISSION LIKE (IDL), and RAPID ALKALINISATION FACTOR (RALF). Secreted peptide hormones are mainly involved in the regulation of plant growth and development. For example, CLE peptides are involved in the regulation of shoot and root apical meristem (SAM and RAM) mataining (Kinoshita et al., 2007; Jun et al., 2010; Katsir et al., 2011), RGF/CLEL/GLV peptides regulate root growth and lateral root formation (Matsuzaki et al., 2010; Meng et al., 2012a; Fernandez et al., 2013), and EPF peptides regulate stomata development (Hara et al., 2007; Hunt and Gray, 2009; Sugano et al., 2010). Most of the secreted peptide hormones are encoded by gene families. In *Arabidopsis*, for example, there are 32 genes encoding CLE peptides, 11 encoding RGF/CLEL/GLV peptides, and six encoding IDL peptides (Sawa et al., 2008; Stenvik et al., 2008; Matsuzaki et al., 2010; Meng et al., 2012a).

Very limited experimental evidence has shown that there is cross-talk between auxin and some of the peptide hormones. For example, PLS and RGF/CLEL/GLV peptides have been shown to regulate auxin transport, and the expression of *PLS* and *RGF*/*CLEL*/*GLV* genes are regulated by auxin (Casson et al., 2002; Chilley et al., 2006; Meng et al., 2012b; Whitford et al., 2012). On the other hand, auxin has been shown to involve in CLE-induced vascular proliferation (Whitford et al., 2008). In this study, we report the identification of *OsCLE48*, a rice *CLE* gene as an auxin response gene, and the functional characterization of *OsCLE48* in *Arabidopsis* and rice.

## Materials and Methods

### Plant Materials and Growth Conditions

The *Arabidopsis thaliana* (*Arabidopsis*) ecotype Columbia (Col-0) and Japonica rice (*Oryza sativa*) variety *Nipponbare* were used for plant transformation. *Arabidopsis* mutant *clv3-2* is in the ecotype *Landsberg erecta* (*Ler*) background (Clark et al., 1995).

Rice seeds were germinated and grown on water for 10 days. Seedlings were then transferred into soil pots and kept in a growth room. *Arabidopsis* seeds were sterilized and grown on plates containing ½ MS (Murashige and Skoog) medium with vitamins (PlantMedia) and 1% (w/v) sucrose, solidified with 0.6% (w/v) phytoagar (PlantMedia). Seedlings were transferred into soil pots and kept in a growth room. For plant transformation and phenotypic analysis of adult plants, *Arabidopsis* seeds were sown directly into soil and grown in a growth room. *Arabidopsis* plants were grown at 20°C, and rice plants at 28°C, with a 16 h light/8 h darkness photoperiod.

### RNA Isolation and RT-PCR

Total RNA from rice was isolated following the procedure described previously for RNA isolation from poplar (Geraldes et al., 2011; Wang et al., 2014). Total RNA from *Arabidopsis* was isolated using the EazyPure Plant RNA Kit (TransGen Biotech) following the manufacturer’s procedure. cDNA was synthesized using the EazyScript First-Strand DNA Synthesis Super Mix (TransGen Biotech) according to the manufacturer’s instructions. RT-PCR or quantitative RT-PCR was used to examine the expression of corresponding genes. Quantitative RT-PCR was performed on a StepOne Real-Time PCR system (Applied Biosystems) with StepOne Software v2.1. All reactions were performed in three replications. Expression of *Arabidopsis* gene *ACTIN2* (*ACT2*) or rice gene *OsACT2* was used as control for RT-PCR. Rice gene *UBQ5* (Jain et al., 2006) was used as control reference gene for quantitative RT-PCR.

All primers used in this study including primers for gene cloning and primers for gene expression analysis were listed in **Table 1**.

**Table 1:** List of primers used in this study.

Primers	Sequences
*OsCLE48-Nde1F*	5^′^-CAACATATGGCGAAGGCGAAGGTTAGC-3^′^
*OsCLE48-Sac1R*	5^′^-CAAGAGCTCTCAGTGATGCTGAGGGTCTGGAC-3^′^
*OsCLE48-qPCRF*	5^′^-TCCTGCTGATTCTCTCGTACT-3^′^
*OsCLE48-qPCRR*	5^′^-TCGTCTCCTCTCGCATTATCT-3^′^
*UBQ5-qPCRF*	5^′^-ACCACTTCGACCGCCACTACT-3^′^
*UBQ5-qPCRR*	5^′^-ACGCCTAAGCCTGCTGGTT-3^′^
*OsCLE48p-Pst1F*	5^′^-CAACTGCAGTTCAAACCGAAATTACGTTC-3^′^
*OsCLE48p-Nco1R*	5^′^-CAACCATGGCCTAGGAAAAACCAAGAGCTC-3^′^
*WUS-Nde1F*	5^′^-CAACATATGGAGCCGCCACAGCATCAGC-3^′^
*WUS-Sac1R*	5^′^-CAAGAGCTCTAGTTCAGACGTAGCTCAAG-3^′^
*CLV3-5*^′^*sequence-Pst1F*	5^′^ -CAACTGCAGCCGGATTATCCATAATAAAAAC-3^′^
*CLV3-5*^′^*sequence-Nde1R*	5^′^ -CCCCCATATGAGAGAAAGTGACTGAGTGAG-3^′^
*CLV3-3*^′^*sequence-Sac1F*	5^′^ -AAAAGAGCTCCCTAATCTCTTGTTGCTTTAA-3^′^
*CLV3-3*^′^*sequence-EcoR1R*	5^′^ -CCCCGAATTCTATGTGTGTTTTTTCTAAACAA-3^′^
*OsACT2-F*	5^′^-TGTATGCCAGTGGTCGTACCAC-3^′^
*OsACT2-R*	5^′^-GAGATGCCAAGATGGATCCTCC-3^′^
*ACT2-F*	5^′^-ATGGATTCGAAGAGTTTTCTG-3^′^
*ACT2-R*	5^′^-TCAAGGGAGCTGAAAGTTGTTTC-3^′^

### Constructs

To generate HA tagged *OsCLE48* construct for plant transformation, the 252 bp full-length ORF of *OsCLE48* was amplified by RT-PCR using RNA isolated from 10-days-old rice seedlings, and cloned in frame with an N-terminal HA tag into the *pUC19* vector under the control of the double *35S* enhancer promoter of *CaMV*. The *pUC19OsCLE48* construct generated was then digested with proper enzymes, and subcloned into vector *pPZP211* and *pCAMBIA1301* to generate binary vector *pPZP211OsCLE48* and *pCAMBIA1301OsCLE48* for *Arabidopsis* and rice transformation, respectively.

To generate the *OsCLE48p-GUS* construct, a fragment that covers the region -1509 to +1 of the start codon of the *OsCLE48* gene was amplified using DNA isolated from rice seedling as template, the PCR products was then used to replace the *PtrCesA8* promoter in the *PtrCesA8p-GUS* construct (Wang et al., 2014). To generate the *CLV3p-OsCLE48* construct, the *35S* promoter and the *nos* terminator in the *pUC19OsCLE48* construct was replaced respectively, by the 1.5-kb 5^′^ upstream and the 1.2-kb 3^′^ downstream regulatory sequences of *CLV3* (Brand et al., 2002). Corresponding constructs in *pUC19* were then digested with proper enzymes, and subcloned into *pPZP211* vector for *Arabidopsis* transformation.

## Plant Transformation

About 5-weeks-old *Arabidopsis* plants with several mature flowers on the main inflorescence were used for plant transformation. The transformation was conducted via *Agrobacterium tumefaciens* (*GV3101*) by using the floral dip method (Clough and Bent, 1998). Phenotypes of transgenic plants were examined in the T1 generation, and confirmed in the following 2–4 generations. For all the transformation, more than five transgenic lines with similar phenotypes were obtained, and at least two lines were used for phenotypic analysis.

Rice transgenic plants were generated by using tissue culture and *Agrobacterium tumefaciens* co-cultivation methods (Hiei et al., 1994).

### Auxin Treatment

To examine the expression of *OsCLE48* in response to auxin, 10-days-old rice seedlings were treated with 10 μM IAA in darkness for 4 h on a shaker at 40 rpm. Samples were frozen in liquid N_2_, RNA was then isolated and used for RT-PCR analysis.

To examine the expression of the *OsCLE48p-GUS* reporter in response to auxin, seedlings of *OsCLE48p-GUS* transgenic *Arabidopsis* plants were treated with 10 μM IAA in darkness overnight on a shaker at 40 rpm. GUS activity was examined by histochemical staining.

### GUS Staining

Seedlings and different organs collected from adult *OsCLE48p-GUS* transgenic *Arabidopsis* plants were stained with X-gluc (5-bromo-4-chloro-3-indolyl-β-D-glucuronide, Rose Scientific Ltd) to monitor GUS activity by following the procedure described by Ulmasov et al. (1997).

### Phylogenetic Analysis

Closely related *Arabidopsis* CLEs to OsCLE48 were identified by BLAST searching *Arabidopsis* proteome database^1^ using the entire amino acid sequence of OsCLE48. Full-length amino acid sequences of OsCLE48 and closely related *Arabidopsis* CLEs were subjected to phylogenetic analysis on Phylogeny^2^ using “One Click” mode with default settings.

## Results

### OsCLE48 is an Auxin Responsive Gene Encoding an A-type CLE

**FIGURE 1:**
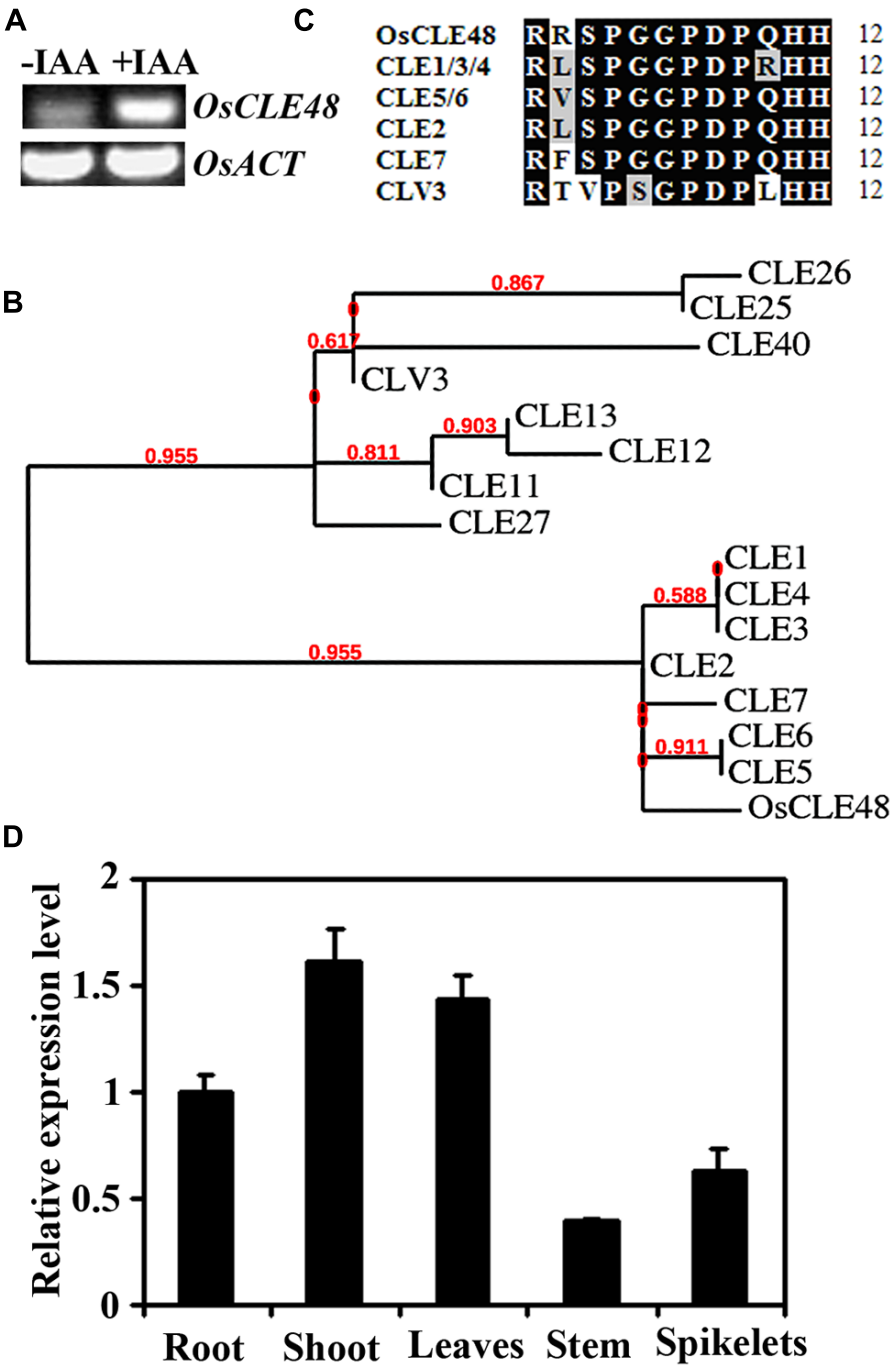
***OsCLE48* is an auxin response gene encoding an A-type CLE peptide. (A)** Expression of *OsCLE48* in response to auxin. Ten-days-old rice seedlings were treated with 10 μM IAA for 4 h, RNA was isolated and RT-PCR was used to examine the expression of *OsCLE48*. Expression of *OsACT2* was used as a control. **(B)** Phylogenetic analysis of OsCLE48 and some of the A-type *Arabidopsis* CLEs. The entire amino acid sequences were used for analysis, and the phylogenetic tree was generated on Phylogeny (www.phylogeny.fr) by using the “One Click” mode with default settings. Branch support values are indicated above the branches. The accession codes for the *Arabidopsis CLE* genes are: *CLV3*, *At2g27250*; *CLE1*, *At1g73165*; *CLE2*, *At4g18510*; *CLE3*, *At1g06225*; *CLE4*, *At2g31081*; *CLE5*, *At2g31083*; *CLE6*, *At2g31085*; *CLE7*, *At2g31082*; *CLE11*, *At1g49005*; *CLE12*, *At1g68795*; *CLE13*, *At1g73965*; *CLE25*, *At3g28455*; *CLE26*, *At1g69970*; *CLE27*, *At3g25905*; *CLE40*, *At5g12990*. **(C)** Sequence alignment of OsCLE48 peptide and closed related A-type *Arabidopsis* CLE peptides. Identical amino acids are shaded in black, and similar amino acids in gray. **(D)** Expression of *OsCLE48* in various tissues and organs of rice. RNA was isolated from roots and shoots of 7-days-old seedlings, and leaves, stem and spikelet of mature plants. Quantitative RT-PCR was used to examine the expression of *OsCLE48*. Rice *UBQ5* was used as reference gene, and expression of *OsCLE48* in root was set as 1. Data represent mean ± SD of three replications.

The expression of some peptide hormone genes such as *PLS* and *RGF*/*CLEL*/*GLV* have been shown to be regulated by auxin (Casson et al., 2002; Chilley et al., 2006; Meng et al., 2012b; Whitford et al., 2012), and auxin has been shown to be involved in CLE-induced vascular proliferation (Whitford et al., 2008), however, no *CLE* genes have been identified as auxin response gene. In an attempt to identify and characterize unknown function auxin response genes in rice, we found that the expression of *OsCLE48* (*LOC_Os05g48730*) was dramatically induced by exogenously application of IAA (**Figure 1A**), a naturally occurred auxin in plants, indicating that *OsCLE48* is an auxin response gene.

*OsCLE48* belongs to the *CLE* gene family in rice. There are at least 47 genes in rice genome encoding CLE proteins. All the CLE proteins have a characteristic amino-terminal signal peptide, and in most of the case, one CLE motif at the carboxyl-terminal (Sawa et al., 2008). By using the entire amino acid sequence of OsCLE48 to BLAST search the *Arabidopsis* protein database^1^, we found that OsCLE48 is most closely related to a subgroup of *Arabidopsis* A-type CLE proteins, CLE1–CLE7. Phylogenetic analysis using the entire amino acid sequences of OsCLE48 and some A-type *Arabidopsis* CLEs showed that OsCLE48 and CLE1–CLE7 formed one subgroup, whereas CLV3 (CLAVATA3) and a few other A-type *Arabidopsis* CLEs formed another subgroup (**Figure 1B**).

Amino acid alignments showed that there is a only one or two aminio acids difference between the 12-amino acid OsCLE48 and CLE1-CLE7 peptides, but four between OsCLE48 and CLV3 peptide (**Figure 1C**).

Quantitative RT-PCR results showed that OsCLE is expressed in all tissues and organs examined. In rice seedlings, *OsCLE48* had relative stronger expression in shoots than in roots. In adult plants, relative higher expression level of *OsCLE48* was observed in leaves, whereas lowest expression was observed in stems (**Figure 1D**).

### Expression and Auxin Response of *OsCLE48* Promoter in *Arabidopsis*

**FIGURE 2:**
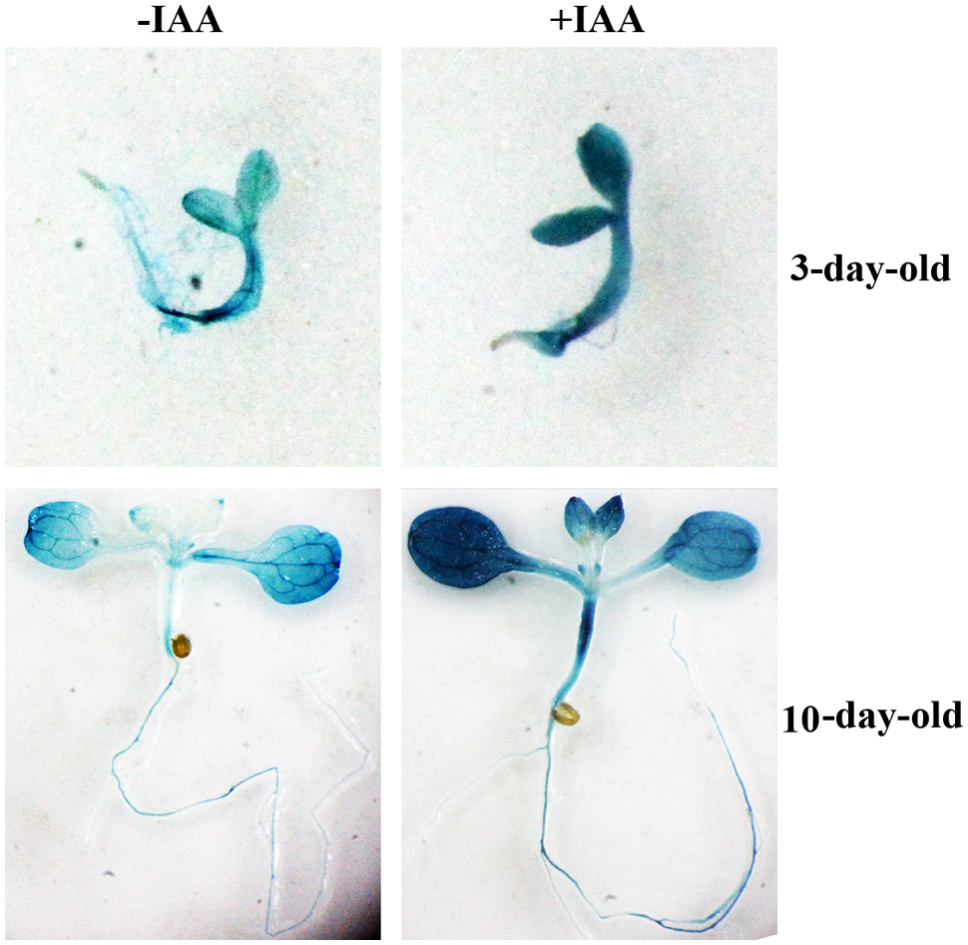
**Auxin response of *OsCLE48p:GUS* reporter gene in *Arabidopsis* transgenic plants.** *Arabidopsis* transgenic seedlings expressing *OsCLE48p:GUS* were treated with 10μM IAA overnight. X-Gluc (5-bromo-4-chloro-3indolyl-β-D-glucuronide) was used for histochemical staining to monitor GUS activity.

None of the *Arabidopsis CLE* genes has been shown to be an auxin response gene. Having shown that *OsCLE48* is an auxin response gene (**Figure 1A**), it encodes a CLE peptide hormone similar to A-type *Arabidopsis* CLEs (**Figures 1B,C**), we wanted to examine if the promoter of *OsCLE48* confers auxin response in *Arabidopsis*. A 1509bp DNA fragment immediately before the start codon of *OsCLE48* gene was used to drive the expression of *GUS* reporter gene in *Arabidopsis*. Three- and 10-days-old transgenic *Arabidopsis* seedlings were treated with exogenously IAA, and GUS activity was examined using X-Gluc as substrates. As shown in **Figure 2**, enhanced GUS staining was observed in seedlings treated with IAA, indicating that the promoter of *OsCLE48* is able to response to auxin in *Arabidopsis*.

**FIGURE 3:**
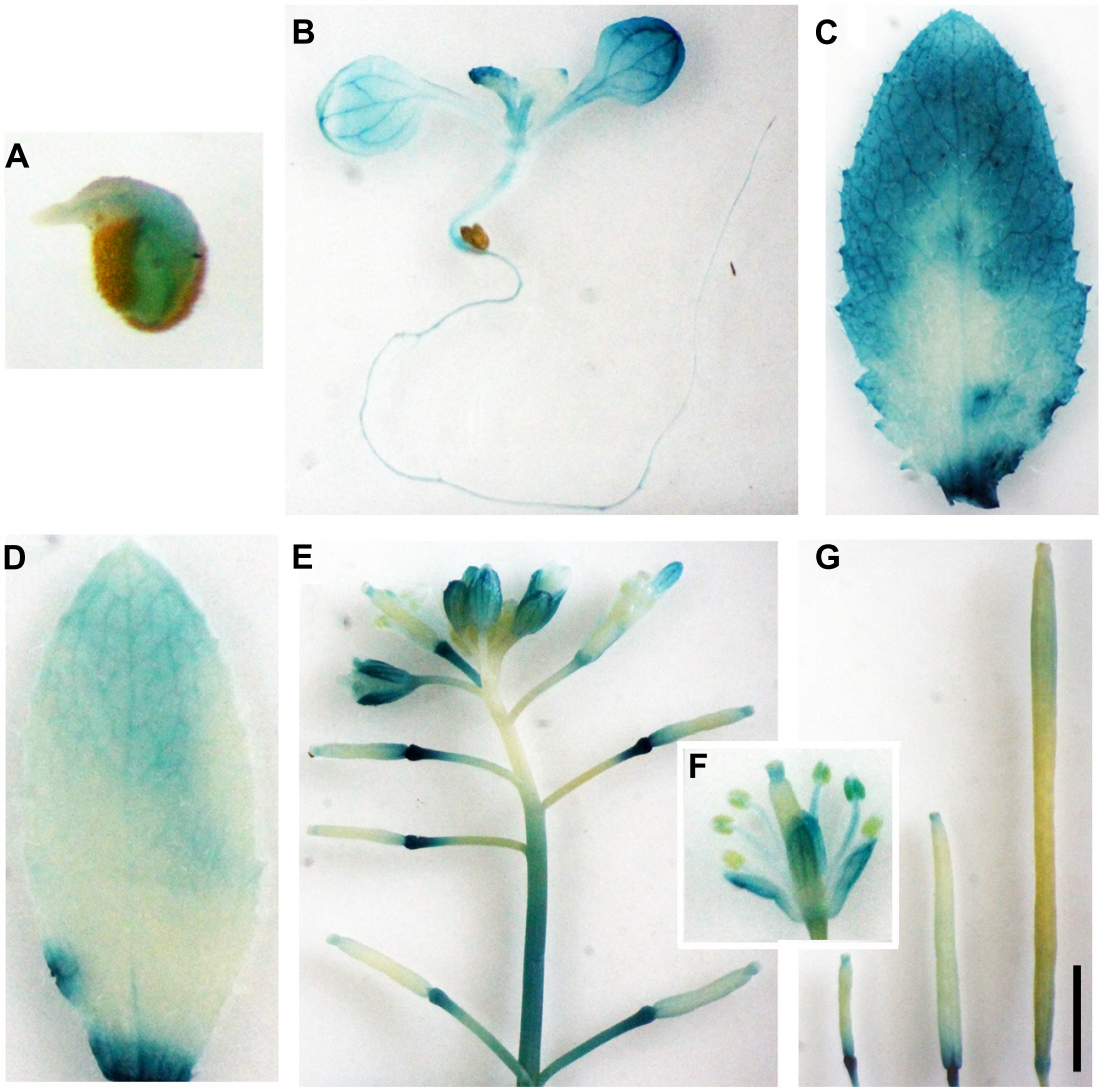
**Expression of *OsCLE48p:GUS* reporter gene in *Arabidopsis* transgenic plants.** Expression of *OsCLE48p:GUS* in germinated seed **(A)**, seedling **(B)**, rosette leaf **(C)**, cauline leaf **(D)**, inflorescence **(E)**, flower **(F)**, and siliques at different developmental stages **(G)** in transgenic *Arabidopsis* plants. X-Gluc was used for histochemical staining to monitor GUS activity. Bar in **(G)**: 3 mm.

Tissue and/or organ specific expression has been observed for most of the *Arabidopsis* A-type *CLE* gene promoters (Jun et al., 2010). To examine if *OsCLE48* promoter is tissue and/or organ specific expressed in *Arabidopsis*, we examine the expression pattern of the *OsCLE48* promoter in *Arabidopsis*. The results showed that GUS activity was detected in germinated seeds, seedlings, and all tissues and organs examined including rosette leaf, cauline leaf, inflorescence, flower, and siliques (**Figure 3**). Expression of the GUS reporter gene in siliques at different growth stages suggest that the expression of *OsCLE48p-GUS* is developmental regulated (**Figure 3G**).

### *OsCLE48* Inhibited Shoot, but not Root Apical Meristem Development in Transgenic *Arabidopsis* Plants

To examine the possible roles of *OsCLE48* in shoot and/or root apical meristem development, we decided to generate *Arabidopsis* transgenic plants expressing *OsCLE48*, and to examine the phenotypes of the transgenic plants.

**FIGURE 4:**
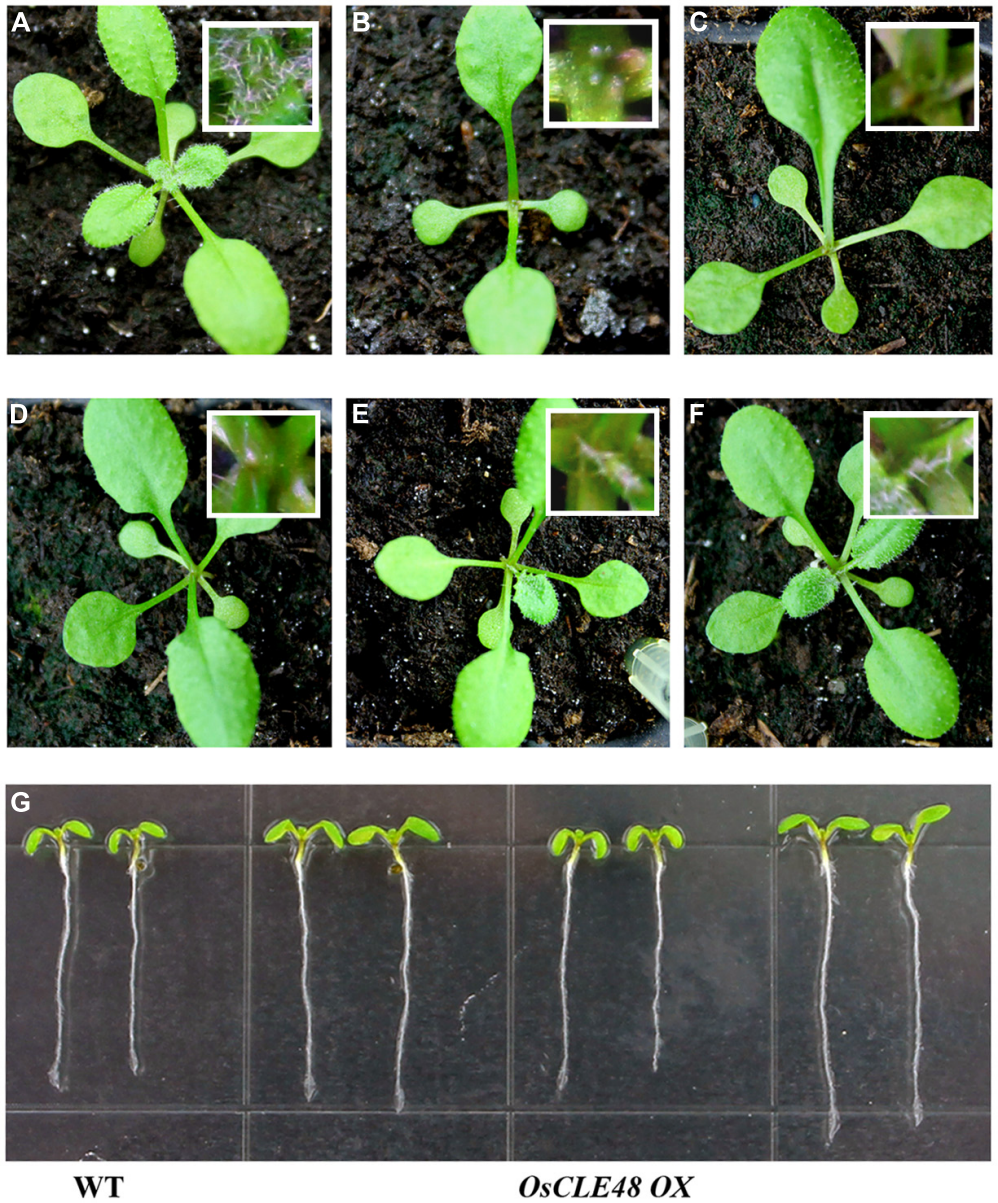
**Phenotypes of transgenic *Arabidopsis* plants expressing *OsCLE48*. (A)** Wild type plant, **(B–F)** transgenic plants with shoot apical meristem arrested at different growth stages. Pictures were taken from 3-weeks-old soil-grown plants. Inserts in **(A–F)**, close views of the shoot apical meristems. **(G)** Seedlings of wild type and transgenic plants (three different lines, two seedlings per line) expressing *OsCLE48*. Pictures were taken from 7-days-old seedlings grown on vertically plates.

*OsCLE48* construct under the control of the *35S* promoter was transformed into *Arabidopsis* Col-0 wild type plants. Multiple lines of transgenic plants with arrested shoot apical meristem were observed. Compared with wild type plant (**Figure 4A**), the shoot apical meristem of the transgenic plants was arrested at different plant growth stages (**Figures 4B–F**). On the other hand, root growth in the transgenic plants are largely unaffected (**Figure 4G**). These results suggest that *OsCLE48* regulates shoot, but not root apical meristem development when expressed in *Arabidopsis*.

### Expression of *OsCLE48* Under the Control of the *CLV3 cis*-Regulatory Elements Rescued the *clv3-2* Mutant Phenotypes

CLV3 is the only *Arabidopsis* CLE identified by mutagenesis, and lost-of-function mutants of *CLV3* gene have enlarged shoot and floral apical meristems with extra floral organs (Clark et al., 1995; Fletcher et al., 1999), whereas all other null mutants identified for other A-type *CLE* genes are largely morphological indistinguishable from wild type plants (Jun et al., 2010). Having shown that expression of *OsCLE48* in *Arabidopsis* resulted in arrested shoot apical meristem (**Figure 4**), a phenotype observed in transgeninc plants overexpressing some of the A-type *CLE* genes (Jun et al., 2010; Katsir et al., 2011), we tested whether *OsCLE48* will rescue the *clv3* mutant phenotypes when expressed under the control of the *CLV3 cis*-regulatory elements in the *clv3-2* mutant.

**FIGURE 5:**
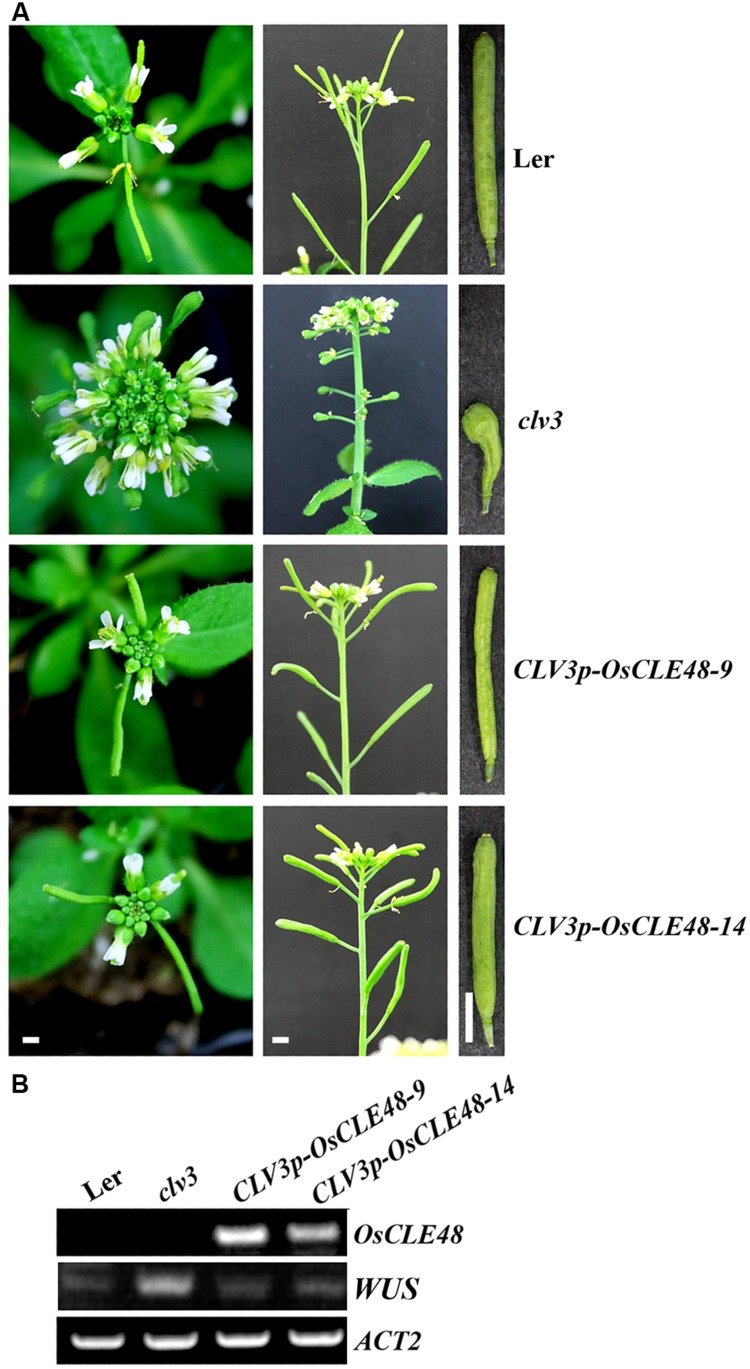
**Rescue of *clv3-2* mutant phenotype by *OsCLE48*. (A)** Inflorescence and silique phenotypes of wild type, *clv3-2*, and *CLV3p-OsCLE48*/*clv3-2* plants. Pictures were taken from 5-weeks-old soil-grown plants. **(B)** Expression of *OsCLE48* and *WUS* in transgenic plants. RNA was isolated from 10-days-old seedlings of wild type, *clv3-2*, and *CLV3p-OsCLE48*/*clv3-2* plants, RT-PCR was used to examine the expression of *OsCLE48*. Expression of *ACT2* was used as a control. Bar: 2 mm, all corresponding photos were taken at same magnification.

The *CLV3p-OsCLE48* construct was generated by replacing the *35S* promoter and the *nos* terminator in the *35S-OsCLE48* construct with the 5^′^ upstream and 3^′^ downstream regulatory sequence of *CLV3* (Brand et al., 2002), respectively. The construct was transformed into *clv3-2* mutants, and phenotypes in the transgenic plants were examined. As described previously (Clark et al., 1995; Fletcher et al., 1999), *clv3-2* mutant plants produce enlarged floral apical meristem, short siliques with increased number of carpels (**Figure 5A**), whereas these phenotypes were almost completely rescued by the entopic expression of *OsCLE48* (**Figure 5A**). The expression of *WUSCHEL* (*WUS*) in the transgenic plants was also restored to wild type level in the *clv3-2* mutant plants expressing *OsCLE48* (**Figure 5B**). These results indicate that *OsCLE48* is functional equivalent to *CLV3* in regulating shoot apical meristem development in *Arabidopsis*.

### Shoot Apical Meristem Development is Largely Unaffected in Transgeinc Rice Plants Overexpressing *OsCLE48*

**FIGURE 6:**
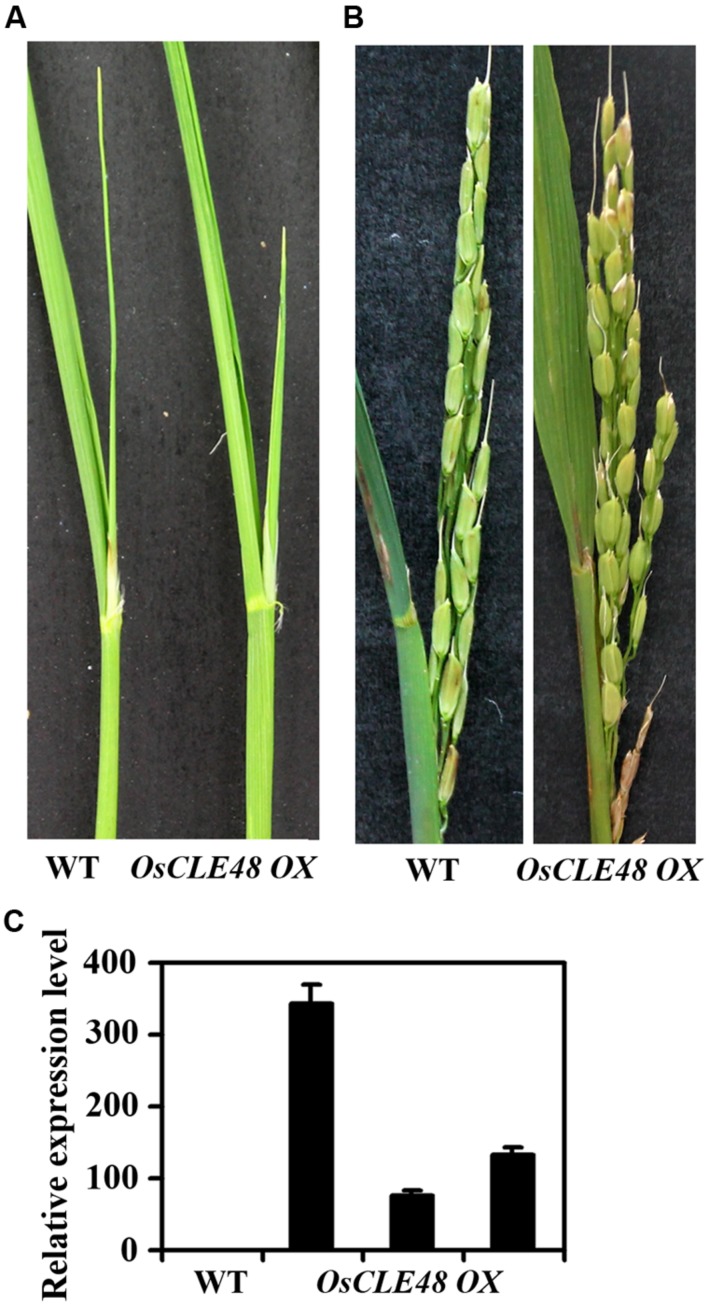
**Phenotypes of rice transgenic plants overexpressing *OsCLE48*. (A)** Shoots and **(B)** Panicles of wild and transgenic rice plants overexpressing *OsCLE48*. **(C)** Expression of *OsCLE48* in wild type and three different lines of transgenic rice plants. RNA was isolated from rice leaves. Quantitative RT-PCR was used to examine the expression of *OsCLE48*. *UBQ5* was used as control reference gene. Expression of *OsCLE48* in wild type was set as 1. Data represent mean ± SD of three replications.

To examine if *OsCLE48* may also play a role in the regulating shoot apical meristem development in rice, we generated transgenic rice plants expressing *OsCLE48* under the control of the *35S* promoter. As shown in **Figure 6A**, shoot development in transgenic rice plants was largely unaffected when compared with wild type plants, and panicles in the transgenic plants was also indistinguishable from that in wild type plants (**Figure 6B**). The overexpression of *OsCLE48* in the transgenic plants was confirmed by quantitative RT-PCR (**Figure 6C**), rolled out the possibility that the morphological similarity observed between transgenic and wild type plants was due to low expression levels of the *OsCLE48* gene.

## Discussion

Plant hormone auxin and peptide hormones have overlapping functions in regulating plant growth and development. Experimental evidence have showed that the expression of some peptide genes such as *PLS* and *RGF/CLEL/GLV* are regulated by auxin (Casson et al., 2002; Chilley et al., 2006; Meng et al., 2012b; Whitford et al., 2012), and auxin is involved in CLE-induced vascular proliferation (Whitford et al., 2008). There are 32 genes in *Arabidopsis* genome, and at least 47 genes in rice genome, encoding CLEs (Kinoshita et al., 2007; Sawa et al., 2008; Jun et al., 2010), however, none of them have been shown to be regulated by auxin. We provide evidence in this study that *OsCLE48* is an auxin response gene, and it regulates shoot apical meristem development when expressed in *Arabidopsis*.

RT-PCR results showed that the expression of *OsCLE48* in rice seedlings was induced by auxin treatment (**Figure 1A**). The expression of the integrated *OsCLE48p-GUS* reporter gene in transgenic *Arabidopsis* was also induced by auxin treatment (**Figure 2**). Sequence scanning showed that there is a TGTCTC auxin response element in the 1509 bp promoter region of *OsCLE48*^3^, it may responsible for the auxin response observed for *OsCLE48* gene. These results indicate the OsCLE48 is an auxin response gene, and its promoter confers auxin responsiveness in *Arabidopsis*. Phylogenic analysis showed that OsCLE48 is closely related to a subgroup of A-type *Arabidopsis* CLEs (**Figure 1B**), and OsCLE48 contains a conserved CLE motif (**Figure 1C**). Taken together, these evidence supports that *OsCLE48* is an auxin response *CLE* gene.

It has been shown that peptide hormones are involved in auxin transport (Chilley et al., 2006; Whitford et al., 2012), and auxin is involved in CLE-induced vascular proliferation (Whitford et al., 2008). We show here that *OsCLE48* is an auxin response gene, thus it may worthwhile to examine if OsCLE48 is involved in regulating auxin transport and/or signaling, in a feed back manner.

Some of the *Arabidopsis* CLE peptide hormones have been shown to regulate shoot and/or root apical meristem maintenance (Kinoshita et al., 2007; Jun et al., 2010; Katsir et al., 2011). Several rice CLEs have also been shown to regulate shoot and root apical meristem development (Chu et al., 2006; Suzaki et al., 2006, 2009). The *OsCLE48-GUS* reporter is expressed in all tissues and organs examined in transgenic *Arabidopsis* (**Figure 3**), a pattern different from that of all the *Arabidopsis CLE* gene promoters tested (Jun et al., 2010). However, expression of *OsCLE48* under the control of the *35S* promoter in *Arabidopsis* arrested shoot apical meristem development (**Figure 4**), a phenotype similar to that observed in *Arabidopsis* transgenic plants overexpressing some of the *CLE* genes (Jun et al., 2010). Expression of *OsCLE48* in the *clv3-2* mutant under the control of the *CLV3* regulatory elements almost completely rescued the *clv3-2* mutant phenotypes (**Figure 5A**). WUS is a key regulatory factor controlling shoot apical meristem stem cell populations. Expression of *WUS* is regulated by CLV3 signaling, in turn, WUS regulates *CLV3* expression in a feedback manner (Brand et al., 2000, 2002; Schoof et al., 2000). Our RT-PCR results showed that the expression of *WUS* in *clv3-2* was restored to wild type level by expressing *OsCLE48* under the control of *CLV3 cis*-regulatory elements (**Figure 5B**). These results suggest that *OsCLE48* encodes a functional CLE peptide hormone. It should note that CLE5/6 is closely related to OsCLE48 (**Figure 1**), however, exogenously application of CLE6/7 peptides does not have any effects in *Arabidopsis* (Kinoshita et al., 2007), possibly because exogenously application of CLE peptides and overexpression of a CLE gene in plant may not always have the same effects.

Several rice CLEs including FLORAL ORGA NUMBER 2 (FON2), FON4, and FON2 SPARE1 (FOS1) have been shown to involve in the regulation of shoot and/or root apical meristem development in rice (Chu et al., 2006; Suzaki et al., 2006, 2009). Both *fon2* and *fon4* mutants have enlarged floral meristem and increased numbers of floral organs (Chu et al., 2006; Suzaki et al., 2006), a phenotype similar to that of *clv3* mutants (Clark et al., 1995; Fletcher et al., 1999). On the other hand, transgenic rice plants overexpressing *FON2* have reduced number of flowers and floral organs (Suzaki et al., 2006), overexpressing of *FOS1* in rice resulted in arrested shoot meristem (Suzaki et al., 2009), exogenous application of *FON4* peptides also resulted in termination of shoot apical meristem (Chu et al., 2006), whereas overexpression of *FON2* in *Arabidopsis* resulted in termination of shoot apical meristem (Suzaki et al., 2006). Our results showed that transgenic rice plants overexpressing *OsCLE48* were morphological indistinguishable from wild type plants (**Figure 6**). We could not role out the possibility that OsCLE48 may regulate shoot apical meristem development in rice without examining lost-of-function mutants. However, there is no loss-of-function mutant available for *OsCLE48* gene. On the other hand, considering that there are at least 47 genes in rice genome encoding CLEs, and there is only one amino acid different between the OsCLE48 peptide and CLE peptides produced by several other CLE genes (Sawa et al., 2008), it is likely that OsCLE48 may have redundant functions with some other CLE genes. Recently, antagonistic peptide technology has been shown to be an efficient way to analyze the functions of some CLEs in *Arabidopsis* (Song et al., 2013), consequently to create antagonistic peptides of OsCLE48 and examine their functions in rice may uncover the roles played by OsCLE48 in rice.

## Conflict of Interest Statement

The authors declare that the research was conducted in the absence of any commercial or financial relationships that could be construed as a potential conflict of interest.

## Footnotes

1 www.phytozome.net

2 www.phylogeny.fr

3 www.phytozome.net
